# Hypovitaminosis D Is Associated with Higher Levels of Inflammatory Cytokines and with HAM/TSP in HTLV-Infected Patients

**DOI:** 10.3390/v13112223

**Published:** 2021-11-04

**Authors:** Elaine Coutinho Netto, Alfredo Carlos Silva, Célia Pedroso, Carlos Brites

**Affiliations:** 1 Sarah Network of Rehabilitation Hospitals, Salvador 41820-900, Brazil; alfredossa@hotmail.com; 2 Laboratório de Pesquisas em Infectologia, Hospital Universitário Edgard Santos (LAPI), Federal University of Bahia, Salvador 40110-060, Brazil; cpedrosoj@gmail.com (C.P.); crbrites@ufba.br (C.B.)

**Keywords:** 25(OH)D, vitamin D, HTLV, Calcitriol, HAM/TSP

## Abstract

Recent studies have shown the effects of vitamin D on host response to infectious diseases. Some studies detected a high prevalence of hypovitaminosis D in HIV-infected patients, but scarce information exists for HTLV-1 infection. We conducted a cross-sectional study to evaluate the frequency of hypovitaminosis D in HTLV-1 patients and its relationship with their immune response in HTLV-infected patients and in age- and gender-matched controls at a Brazilian rehabilitation hospital. We compared vitamin D, interleukin-6 (IL-6), tumoral necrosis factor-alpha (TNF-α) and interferon-gamma (IFN-γ) levels across groups. Logistic regression was utilized to assess the association between hypovitaminosis D and cytokine levels. We enrolled 161 HTLV-infected subjects (129 HTLV-associated myelopathy/tropical spastic paraparesis (HAM/TSP) patients, 32 asymptomatic HTLV carriers) and equal number of HTLV-negative controls. We observed a significantly higher prevalence of hypovitaminosis D in patients with HAM/TSP than in HTLV asymptomatic carriers (*p* < 0.001), or controls (*p* < 0.001). HAM/TSP patients also had higher levels of IL-6 and IFN-γ than asymptomatic carriers. Patients with HAM/TSP and hypovitaminosis D had higher levels of TNF-α than asymptomatic HTLV carriers. These findings suggest hypovitaminosis D plays a role in HAM/TSP pathogenesis, and it needs to be evaluated in further studies.

## 1. Introduction

The importance of vitamin D in bone metabolism is well known, and the role of vitamin D deficiency in two major disorders of calcium metabolism (rickets in children and osteomalacia in adults) was recently recognized [1,2]. The expression of vitamin D receptors (VDR) in different organs (pancreas, brain, muscles, adipose tissue, colon, breast and immune cells) reinforces its importance in non-bony processes [3]. Many studies have linked vitamin D status to autoimmune diseases, type 1 diabetes mellitus, cardiovascular diseases, cancer and infections [1,2,3,4]. It has been estimated that more than 1 billion people have either 25-hydroxyvitamin D (25(OH)D) deficiency or insufficiency [2]. As a result, routine screening for low 25 (OH) D levels and supplementation of vitamin D has become increasingly common [1,2,3,4,5].

Immune system cells express VDR, which converts 25(OH)D, by enzymatic reaction, to the active form of vitamin D, the 1,25-dihydroxyvitamin D (1,25(OH)_2_D), modulating and enhancing the immune response to infectious diseases [1,2,3]. The 1,25(OH)_2_D suppresses T cell activation and genes involved in cell proliferation and differentiation, and downregulates the production of proinflammatory cytokines such as interferon-gamma (IFN-γ), tumor necrosis factor-alpha (TNF-α), interleukin-2 (IL-2) and interleukin-12 (IL-12) [5,6,7,8,9]. It can shift the activated T cell response from a Th1 to a Th2-like response and can play an important role on antimicrobial defense and immune regulation, including monocyte chemotaxis and differentiation into macrophages, nitric oxide production by macrophages, and production of cathelicidin and beta defensin-4, antimicrobial peptides whose functions include inhibition of viral replication [9].

Because of vitamin D’s critical anti-inflammatory and antimicrobial functions, vitamin D deficiency has been associated to several infectious diseases, including HIV [10,11,12,13].

Human T lymphotropic virus type 1 (HTLV-1) was the first human retrovirus identified. The majority of infected individuals remain lifelong as asymptomatic carriers (AC). Approximately 0.2% to 3.8% of HTLV infected individuals develop a progressive neurological disease named HTLV-associated myelopathy/tropical spastic paraparesis (HAM/TSP) and 2% to 5% develop an aggressive mature T cell malignancy, the adult T cell leukemia/lymphoma (ATL) [14]. HAM/TSP is a chronic, inflammatory disease of the central nervous system and is characterized by slowly progressive spastic paraparesis, lower limb sensory disturbance and bladder/bowel dysfunction [15,16]. Saito et al. found that the VDR ApaI polymorphism is associated with increased risk of HAM/TSP, although this polymorphism does not influence the proviral load of HTLV-1 in HAM/TSP patients or asymptomatic carriers [17]. Immunomodulatory actions of 1,25 (OH)_2_D are mediated by its interaction with the VDR receptor, which is expressed on the surface of activated and non-activated lymphocytes [17].

Although the role of the immune response in HTLV pathogenesis is not fully understood, it seems that the efficacy of immune response in controlling viral persistence determines the outcome of HTLV-associated diseases. The available data suggest that the interaction between virus and host defense mechanisms plays a critical role in determining the risk of development of HTLV-associated diseases among HTLV carriers [18,19,20].

To the best of our knowledge, there is only one published study describing an association between hypovitaminosis D and HAM/TSP patients, coupled with some lipid abnormalities, but without information on cytokines production [21]. The present study aims to evaluate the frequency of hypovitaminosis D and its relationship with clinical status and immune response in patients infected with HTLV.

## 2. Materials and Methods

### 2.1. Study Design and Population

We performed a cross-sectional study in the period of April 2014 to November 2016 at a rehabilitation hospital (Sarah Network of Rehabilitation Hospitals) in Salvador, Northeast Brazil. Cases of HAM/TSP or asymptomatic carriers (AC) were diagnosed according to criteria of the World Health Organization (WHO) [22]. Infection by HTLV-1/2 was confirmed by positive HTLV serology (ELISA and Western blot) in serum and cerebrospinal fluid (for HAM/TSP patients). They were consecutively recruited at Sarah’s HTLV clinic. We randomly selected HTLV-negative patients attended at the same hospital and 38 hospital staff members to serve as controls, using electronic medical records. Controls were matched by sex and age.

Individuals reporting use of vitamin D, anticonvulsants or corticosteroids, and patients with moderate to severe chronic renal failure, were not eligible to participate in the study.

The Hoffer Functional Ambulation Scale was used to evaluate the functional gait level of individuals and to classify them as community ambulators (level 1), household ambulators (level 2), non-functional ambulators (level 3) and non-ambulators (level 4) [23].

### 2.2. Laboratory Assays

Sera from all cases were screened for HTLV 1/2 antibodies by EIA (Enzyme-Linked Immunosorbent Assay HTLV-1/2, Symbiosys, São Paulo, Brazil). Confirmation and discrimination between HTLV-1 and HTLV-2 was achieved by Western blot (HTLV Blot 2.4, MP Biomedicals Asia Pacific Pte LTD. Singapore).

Plasma concentration of 25(OH)D was measured by ELFA. We used the current Institute of Medicine’s (IOM) [22] and Endocrine Society’s [23] guidelines to define vitamin D deficiency as a plasma 25(OH)D concentration < 20 ng/mL, vitamin D insufficiency as a plasma 25(OH)D concentration ≥ 20 and <30 ng/mL, and normal vitamin D status as a plasma 25(OH)D concentration ≥ 30 ng/mL. In 2018, The Brazilian Society of Clinical Pathology/Laboratory Medicine (SBPC/ML) and the Brazilian Society of Endocrinology and Metabolism (SBEM) redefined the ideal 25(OH)D_3_ values for the population and should be stratified according to age and individual clinical characteristics following the normal cut-off point of 25(OH)D_3_ ≥ 20 ng/mL for the population healthy adult up to 60 years of age [24,25].

HTLV-1 proviral load was performed using the Real-Time Polymerase Chain Reaction (RT-PCR) for detection of HTLV-1 pol gene and exon 12 of the human albumin gene [26].

Plasma levels of proinflammatory cytokines (IL-2 and IL-6, TNF-α, IFN-γ-) were measured by ELISA (Human IFN-y DuoSet catálogo #285-05; Human IFN-a DuoSet kit catálogo#DY210-05; Human IL-2 DuoSet kit catálogo #DY202-05; Human IL-4 DuoSet kit catálogo#DY204-05;Human IL-6 DuoSet kit catálogo #DY206-05; Human IL-10 DuoSet kit catálogo#DY217B-05,R&D Systems, Minneapolis, MN, USA), in samples collected and stored at −80 °C until analysis. Laboratory personnel were blinded to clinical information.

### 2.3. Statistics Analyses

The sample size was calculated using the prevalence (49%) of hypovitaminosis D in the general population attended at the same hospital by August 2016. The sample size was estimated in 127 individuals for each group to have 80% power using 95% confidence intervals. Significance difference was set as 0.05.

Simple frequency distributions of the variables, means and measures of dispersion (standard deviation [SD]) for continuous variables with normal distribution were calculated. For variables with non-normal distribution the median and interquartile range (IQR) were used. If the distribution was not normal, the Kruskal–Wallis test was used. To confirm the observed differences, we used a post-test for multiple comparisons (Dunn Test with Bonferroni’s correction). Categorical variables were compared by chi-square test.

All analyses were carried out using SPSS Statistical version 18.0 (SPSS Inc. Chicago, IL, USA).

### 2.4. Ethical Considerations

All enrolled subjects provided written informed consent prior to study participation. The study was approved by the Research Ethics Committee at the Sarah Network of Rehabilitation Hospitals (Plataforma Brasil CAAE: 1119331240000 0022, July 2014).

## 3. Results

The study population comprised 161 HTLV-infected subjects, 129 of them (80.1%) had HAM/TSP and 32 (19.9%) were AC. They were matched by sex and age to 161 HTLV-negative controls. Controls included patients with a diagnosis of stroke’s sequelae (36.6%), hospital’s healthy professionals (23.6%), traumatic spinal cord injury sequelae (7.5%), Parkinson’s disease (5%), ataxia (3.7%), idiopathic myelitis sequelae with negative serology for HTLV (3.7%), multiple sclerosis (2.5%), traumatic brain injury sequelae (1.9%), and other diagnosis (15.5%). Most (95%) of the participants were black or racially mixed. Table 1 summarizes patients’ characteristics.

Difference in sun exposure is a well-known confounding variable associated with risk factors for hypovitaminosis D. Patients with greater neurological impairment are likely to be less exposed to sunlight. To rule out the effect of sunlight exposure on vitamin D levels, we compared the vitamin D levels for patients with the same locomotion capacity, as measured by Hoffer scale. There was a significant difference in mean vitamin D levels between community ambulators HAM/TSP patients (27.5 ± 9.8 ng/mL) and community ambulators controls (31.2 ± 9.8 ng/mL, *p* = 0.015) as well as between wheelchair-restricted HAM/TSP patients (20.6 + 9.5 ng/mL) and controls with same mobility condition (27 ± 10.4 ng/mL, *p* = 0.001). Most patients were classified as community ambulators in both, HAM/TSP (57.3%) and control group (60%).

HAM/TSP patients showed a significantly higher frequency of hypovitaminosis D than AC (*p* < 0.001) or controls (*p* < 0.001). Table 2 summarizes the mean levels of vitamin D for the study’s groups.

Patients in HAM/TSP group had a higher mean proviral HTLV load (5941 SD: 14,038 copies of HTLV-1/10^5^ cells/mL) compared to AC (508 SD: 1.038 copies of HTLV-1/10^5^ cells copies/mL, *p* = 0.03; 95% CI: 267; 10,597).

**Figure 1:**
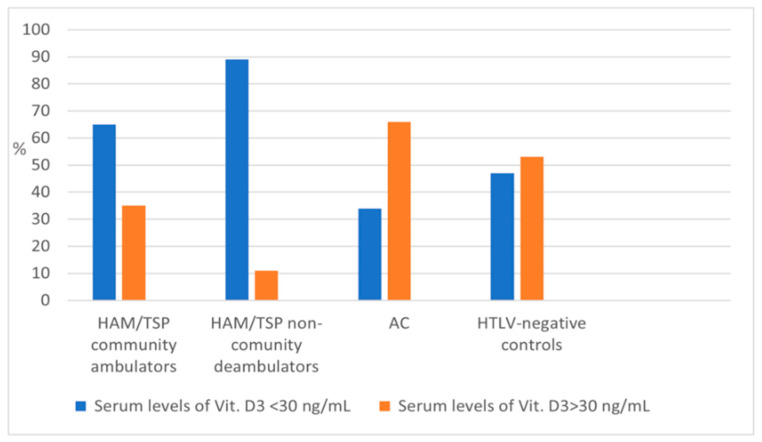
Proportion (%) of subjects with normal/abnormal vitamin D levels according to HTLV status and ambulation capacity.

**Figure 2:**
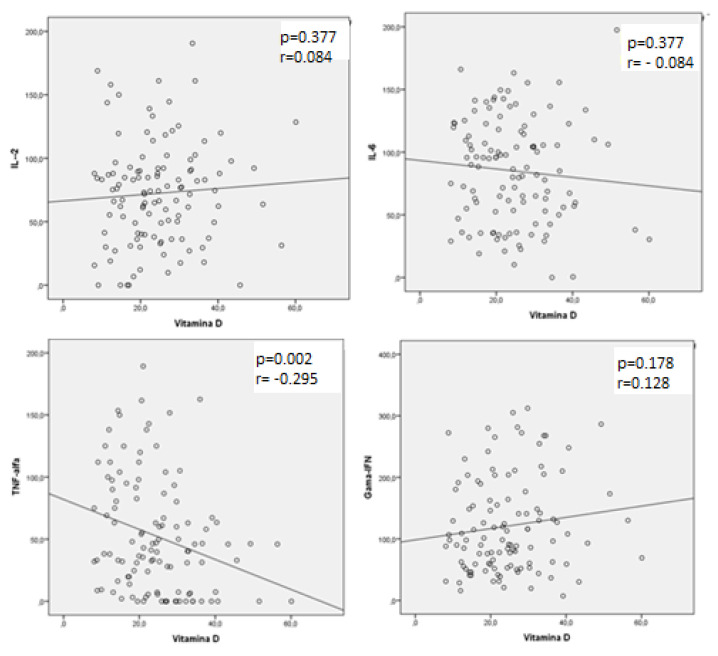
Correlation between serum levels of vitamin D and pro-inflammatory cytokines (IL-2, IL-6, TNF-α and IFN-γ) in HAM/TSP patients.

**Figure 3:**
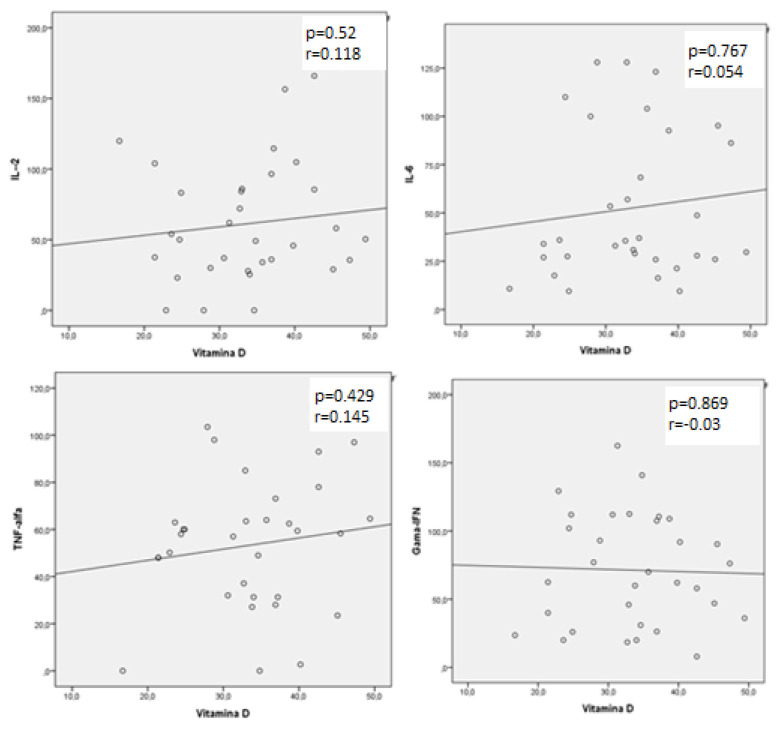
Correlation between serum levels of vitamin D and pro-inflammatory cytokines (IL-2, IL-6, TNF-α and IFN-γ) in HTLV-asymptomatic carriers.

HTLV-infected patients with hypovitaminosis D had significantly higher levels of IL-6 (95 pg/mL; IQR: 36–119) and TNF-α (50 pg/mL; IQR: 24.3–94.2) than patients with normal vitamin D levels (IL-6: 57 pg/mL; IQR: 31.4–99; *p* < 0.001; TNF-α: 40.3 pg/mL, IQR: 6.8–62.8; *p* = 0.001). A post-test (Dunn’s test with Bonferroni’s correction) confirmed the significant differences between HAM/TSP and AC groups. Table 3 shows the cytokine levels according to patient group. In HAM/TSP patients, a significant negative correlation was detected between TNF-α and vitamin D levels (r = −0.272; *p* = 0.009). Figure 1, Figure 2 and Figure 3 show these findings. Values for cytokines levels are also depicted in Table 4.

## 4. Discussion

To the best of our knowledge, this is the first study to demonstrate an association between hypovitaminosis D and immune response in this population. We found a higher prevalence of hypovitaminosis D in HAM/TSP patients than in controls and AC and a significant association between vitamin D deficiency and higher levels of TNF-α in HAM/TSP patients, but not in AC. Our findings suggest vitamin D levels play a role in pathogenesis of HTLV infection.

Reports on vitamin D levels in patients with HTLV infection are scarce, but there is consistent evidence on its role in HIV infection and the regulation of immune response to HIV [10,11,12,13,27,28,29,30,31,32]. The effects of 1,25(OH)_2_D are mediated through its interaction with VDR, allowing its access to the nucleus where its binds to vitamin D response elements (VDRE) and regulates gene transcription. VDR expression is also found in immune system cells, like monocytes, macrophages, dendritic cells, natural killer cells, and T and B cells, which supports its immunomodulatory effects [5,6,7,8,9].

Vitamin D effect on viral expression was associated with the cytokine environment, since vitamin D enhanced HIV expression in the presence of TNF-α but inhibited viral expression in the presence of IFN-γ, IL-6 and granulocyte–macrophage colony-stimulating factor (GM-CSF) [33]. In addition, vitamin D is involved in T cell activation and phenotype modulation as well as in antigen-presenting cells function and IL-10 synthesis [34]. It a direct correlation between HIV viral load and inflammation biomarkers was also observed, but not between vitamin D status and current CD4 counts [35,36,37].

Spontaneous cytokine production is seen in both AC and HAM/TSP patients [37]. HAM/TSP patients show high frequencies of Tax-specific CD8+ T cells in peripheral blood and cerebrospinal fluid, high antibody titers to HTLV and increased production of proinflammatory cytokines (IFN-γ, TNF-α, interleukin-1beta (IL-1 β), IL-6 and IL-12) [17,37]. In our study, we observed high levels of IFN-γ and IL-6 in the HAM/TSP group. In addition, higher levels of TNF- α were observed in the HAM/TSP group presenting with hypovitaminosis D, than in patients with normal levels of vitamin D. TNF-α may induce a defect in the hydroxylation and activation of vitamin D by blocking the stimulation of parathyroid hormone (PTH) on 1-α-hydroxylase. TNF-α levels are increased in HIV patients due to the status of immune hyperactivation and may be the cause of hypovitaminosis D in such individuals [38,39]. TNF-α levels are also consistently increased in HAM/TSP patients [40,41,42]. The most consistent theory on mechanisms of HAM/TSP pathogenesis indicates that neuronal damage is caused by a bystander effect of activated CD8 + T lymphocytes against CD4 + T cells infected by HTLV. This response is mediated by production of high levels of proinflammatory cytokines (TNF-α and IFN-γ) and decreased levels of IL-4 and IL-10 [43]. The association between hypovitaminosis D and increase in levels of TNF-α in patients with HAM/TSP may suggest vitamin D is involved in the pathogenesis of neurological disease caused by HTLV-1.

Hoe et al. examined the effects of 1,25(OH)_2_D on peripheral blood mononuclear cells (PBMCs) and purified immune cell subsets isolated from healthy adults. They found that 1,25(OH)_2_D significantly reduced proinflammatory cytokines like TNF-α, IFN-γ, and IL-1β as well as the chemokine IL-8 for both ligands, while levels of IL-10 were increased. Levels of IFN-γ were significantly higher in adults with insufficient levels of vitamin D than in those with normal vitamin D levels. These results suggest that 1,25(OH)_2_D is an effective modulator of the inflammatory response [44]. Cantorna et al. also suggested an important role of 1,25(OH)_2_D in regulating T cells to limit immune mediated diseases [45].

To date, there are few reports in literature on the frequency of hypovitaminosis D in patients infected with HTLV, and they did not focus on the potential association between vitamin D levels and patients’ immune response [17,21]. Our results indicate that vitamin D levels can play a role in HAM/TSP pathogenesis.

Our study has some limitations, like its cross-sectional design which does not allow us to define causality. Variability in vitamin D levels due to sunlight exposure or dietary differences could be a potential confounding factor, but the use of matched controls makes our results consistent and strengthen the detected associations. We suggest that our results are strong enough to indicate the need of additional research to define the role of vitamin D in the pathogenesis of HTLV infection.

Once hypovitaminosis D is associated with negative outcomes of some infectious diseases, like sepsis, HIV, COVID-19, tuberculosis and respiratory infections, and there is increasing evidence about its role on the immune response, clinicians should consider routine screening of HTLV-infected patients for vitamin D insufficiency/deficiency [46,47]. Vitamin D supplementation is a cheap and safe intervention and could be recommended for patients with HTLV-1 infection in an attempt to improve their immune balance.

## Figures and Tables

**Table 1:** Demographic characteristics of patients infected by HTLV and HTLV-negative controls.

Patients Characteristics	HAM/TSP N = 129 (%)	HTLV-Asymptomatic Carriers N = 32 (%)	HTLV-Negative Controls N = 161 (%)	*p*-Value
**Gender** Male Female	38 (29.5) 91 (70.5)	8 (25) 24 (75)	46 (28.6) 115 (71.4)	0.88
**Age in Years (Mean ± SD)**	51.6 ± 13.6	48.5 ± 13.4	51.6 ± 13.5	0.58

**Table 2:** Comparison between serum levels of vitamin D between the HAM/TSP, HTLV-asymptomatic carriers and controls groups.

Variables	HTLV+ (N = 161) ^1^	HAM/TSP (N = 129) ^2^	Asymptomatic Carriers HTLV ^3^ (N = 32)	Controls (N = 161)
Mean 25(OH)D_3_ * ng/mL (SD) **25(OH)D <20** (%) ** 25(OH)D ≥ 20 (%) ** 25(OH)D < 30 (%) *** 25(OH)D ≥ 30 (%) ***	26.3 (10.5) 47 (29.2%) 114 (70.8%) 105 (65.3%) 56 (34.7%)	24.5 (10.3) 46 (35.7%) 83 (64.3%) 94 (72.9%) 35 (27.1%)	33.5 (8.3) 1 (3.1%) 31 (96.8%) 11 (34.4%) 21 (65.6%)	29.5 (10.3) 30 (18.6%) 131 (81.4%) 82 (51%) 79 (49%)
**Mean proviral HTLV load** (copies of HTLV-1/10^5^ cells/mLSD )	-	5.94 ± 14.04	508 ± 1.04	-

* Student’s *t* test. ^1^ *p* = 0.007 (CI95%: −5.43; −0.86)-HTLV vs. controls. ^2^ *p* < 0.001 (CI95%: −12.8; −5.06)-HAM/TSP vs. HTLV-asymptomatic carriers. *p* < 0.001 (CI95%: −7.31;−2.52)-HAM/TSP vs. controls. ^3^ *p* = 0.03 (CI 95%: 0,19; 7,83)-HTLV-asymptomatic carriers vs. controls. ** cut-off of 20 ng/mL (chi-square test). ^1^ *p* = 0.026 (CI 95% 0.329; 0.936)-HTLV vs. controls. ^2^ *p* < 0.001 (CI95%: 0.008; 0.44)-HAM/TSP vs. HTLV-asymptomatic carriers. *p* = 0.001 (CI95%: 0.242; 0.706)-HAM/TSP vs. controls. ^3^ *p* = 0.029 (CI 95%: 0.932; 54.07)-HTLV-asymptomatic carriers vs. controls. *** cut-off of 30 ng/mL (chi-square test). ^1^ *p* = 0.009 (CI 95% 0.354; 0.866)-HTLV vs. controls. ^2^ *p* < 0.001 (CI95%: 0.085; 0.44)-HAM/TSP vs. HTLV-asymptomatic carriers. *p* < 0.001 (CI95%: 0.235; 0.635)-HAM/TSP vs. controls. ^3^ *p* = 0.087 (CI 95%: 0.897; 4.37)-HTLV-asymptomatic carriers vs. controls.

**Table 3:** Comparison between serum levels of proinflammatory cytokines (IL-2, IL-6, TNF-α and IFN-γ) between the HAM/TSP patients and HTLV-asymptomatic carriers.

Serum Levels of Pro-Inflammatory Cytokines (pg/mL)	HAM/TSP	HTLV-Asymptomatic Carriers	*p*-Value
**IL-2**	74.1 (39.9–92.1)	50.2 (31–85.8)	0.121 ^1^
**IL-6**	87.8 (52.7–117.3)	34.8 (26.2–91)	**<0.001 ^2^**
**TNF-α**	41.2 (8.1–81.5)	58.1 (31.4–64.4)	0.341 ^3^
**IFN-γ**	98.2 (58.6–171)	66.2 (32.2–108.6)	**0.001 ^4^**

Median and interquartile range (IQR). Confidence interval (IC): 95% (IC95%). Wilcoxon-Mann–Whitney test. ^1^: U = 1482.5; *p* = 0.121. ^2^: U = 973.5; *p* < 0.001 (r = −0.33 IC95% [−0.18; −0.47]). ^3^: U = 1608.5; *p* = 0.341. ^4^: U = 1123; *p* = 0.001 (r = −0.27 IC95% [−0.11; −0.42]).

**Table 4:** Comparison between serum levels of proinflammatory cytokines (IL-2, IL-6, TNF-α and IFN-γ) between the HAM/TSP patients and HTLV-asymptomatic carriers.

Serum Levels of Pro-Inflammatory Cytokines (pg/mL)	HAM/TSP	HTLV-Asymptomatic Carriers	*p*-Value
IL-2	74.1 (39.9–92.1)	50.2 (31–85.8)	0.121 ^1^
IL-6	87.8 (52.7–117.3)	34.8 (26.2–91)	<0.001 ^2^
TNF-α	41.2 (8.1–81.5)	58.1 (31.4–64.4)	0.341 ^3^
IFN-γ	98.2 (58.6–171)	66.2 (32.2–108.6)	0.001 ^4^

Median and interquartile range (IQR). Confidence interval (IC): 95% (IC95%). Wilcoxon-Mann–Whitney test. ^1^: U = 1482.5; *p* = 0.121. ^2^: U = 973.5; *p* < 0.001 (r = −0.33 IC95% [−0.18; −0.47]). ^3^: U = 1608.5; *p* = 0.341. ^4^: U = 1123; *p* = 0.001 (r = −0.27 IC95% [−0.11; −0.42]).

## Data Availability

The data presented in this study are available on request from the corresponding author. The data are not publicly available due to institutional policy.
