# Correction: Netto et al. Hypovitaminosis D Is Associated with Higher Levels of Inflammatory Cytokines and with HAM/TSP in HTLV-Infected Patients. *Viruses* 2021, *13*, 2223

**DOI:** 10.3390/v14081633

**Published:** 2022-07-26

**Authors:** Elaine Coutinho Netto, Alfredo Carlos Silva, Célia Pedroso, Carlos Brites

**Affiliations:** 1 Sarah Network of Rehabilitation Hospitals, Salvador 41820-900, Brazil; alfredossa@hotmail.com; 2 Laboratório de Pesquisas em Infectologia, Hospital Universitário Edgard Santos (LAPI), Federal University of Bahia, Salvador 40110-060, Brazil; cpedrosoj@gmail.com (C.P.); crbrites@ufba.br (C.B.)

## Error in Figure

In the original publication [1], there was a mistake in the Figure 1 labels of the vertical axis, which were not properly formatted: The values in the vertical axis were shown as RAL1900, due to an error in the figure’s uploading process. The correct values are 0, 20, 40, 60, 80 and 100.

The corrected Figure 1 appears below. The authors apologize for any inconvenience caused and state that the scientific conclusions are unaffected. The original publication has also been updated. 

## Figures and Tables

**Figure 1:**
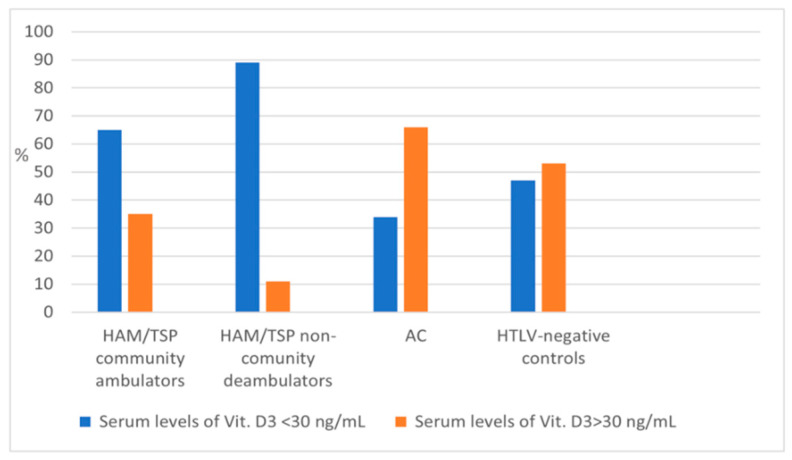
Proportion (%) of subjects with normal/abnormal vitamin D levels according to HTLV status and ambulation capacity.
